# Examining Boards: An Open Letter

**Published:** 1897-11

**Authors:** B. F. Arrington

**Affiliations:** Goldsboro, N. C.


### EXAMINING BOARDS.—AN OPEN LETTER.

Dr. B. Holly Smith, Baltimore, Md.:

Dear Doctor,—I have read carefully and with much interest the Pennsylvania act you furnished me for perusal, and will avail myself of this, the earliest opportunity, to express to you my convictions on the subject. I must be frank with you, and will say in all candor that my views and feelings are not in sympathy with the wording of the law as a whole.

The first and one very serious objection is this, that it will tend to strengthen and perpetuate dental examining boards beyond, in all probability, a period of usefulness, and the reform and good they were designed to accomplish in the interest of dentistry as a profession and suffering humanity.

When dental examining boards were created it was deemed essential, as a stimulus to dental college faculties, to be more exacting and stringent with candidates for graduation, that they might send graduates forth to practise better qualified than by the prevailing custom.

That in part has been accomplished, and the evidences are very perceptible and gratifying. The good work accomplished justifies hope for future development of progressive good results on a higher plane of action. So perceptible is the improved work in college laboratories, operating- and clinic-rooms, that the question now arises, Is there further need of such organizations? May not abuses be indulged and harm rather than good come to the profession at large?

Holding to the views I do, believing it to be the duty of dental college faculties to prepare students for graduation and practice for the benefit and satisfaction of the public, I also honestly believe that dental college faculties, as a whole, are infinitely more competent and better prepared to judge of the qualifications requisite for successful practice, and ultimate rendering of good service to the honor and credit of the profession, than are the majority of dentists who compose dental examining boards.

With these convictions I cannot advise nor sanction the continuance of such organizations. So far they have served a reasonably good purpose, have aroused thought and action, and doubtless have stimulated college faculties to a livelier sense of the distinguished responsibilities that rest upon them, and to the extent that it is now recognized and realized fully that they are doing more thorough work in course of preparation of dental students for graduation and successful practice, and in all probability will increase in united effort for completeness of work that will fully meet the general demand. In the course of a few years the universal college curriculum will be such as to satisfy perfectly the profession and the public, and there will be general proclaim that there is no further need of examining boards, and that a diploma from any accredited dental college in this country shall permit the possessor to open office and practise without question or hinderance in any State or special locality within our broad domain of territory.

Then examining boards, after having done some good (for which credit is due) and dispensed considerable injustice, will be a thing of the past. At present, under the existing state of things, colleges are weakening, and any one of these at any time is liable to be brought into disrepute by one prejudiced, scheming manipulator on an examining board. There are good men and competent on these boards, but they do not always have the control. Not only colleges are liable to suffer injustice, but young graduates of true worth and fair attainments experience unjust treatment and have no redress.

Men selected to compose the boards are not always the most competent and best suited in all respects for the honorable position, and such being the case, the ruling is as apt to be for evil as for good, as actions are not always based upon strict principles of equity and justice. It is not unfrequent that young graduates of true merit and well prepared to begin practice are rejected, and there is no immediate means for reversal of decision, and the humiliation and mortification must be endured for a year at least.

In some States for several years past a rejection by examining boards of one-fifth or more of all applicants for license to practise has prevailed, and so it is with medical examining boards. This is unreasonable and wrong, and sometimes criminally unjust, and should be checked and righted, and the sooner it is done the better for the elevation and dignity of the dental profession. If the present order of things is not dealt with and checked there will soon be a marked retrogression in both professions.

State dental schools, appendages to universities and colleges, with chartered rights and privileges and very few advantages, will be the order of the day with the dental, as is now prevalent and rapidly increasing with the medical profession, and very naturally a weak state of things will follow, with no upward tendency whatever.

The creation of examining boards was designed for general good, strengthening and advancing the profession in public estimation, and to "guard the public" (so proclaimed) against empiricism and incompetent practitioners,—motives very commendable, but almost a feature of too much unselfishness to continue and prove effective and beneficial. We are all by nature more or less selfish, and when latitude is given, as in the case of examining boards, the selfish feature will assert itself occasionally, and sometimes it is difficult to keep it in check, as evidenced by expressions like these: "We must put on the brakes and stop this wholesale graduating; the whole country will soon be flooded with young dentists; there are too many now." What meaneth such expressions? I pronounce them unworthy of the source from which they emanated, and are weakening to organizations designed for good.

Time has developed the fact that the good for which examining boards were designed has not been accomplished in full, and in all probability never will be. Purifying is requisite, and the remedy is not in sight to justify contention for further existence of these. If the present course be persisted in much longer it will, in my judgment, prove seriously pernicious, and dental colleges will be regarded as of but little importance, and any faculty will be subject to the prejudices and whims of undeserving, domineering men on these boards.

All must confess, and it is to be regretted, that some dental colleges (owing chiefly, I believe, to the great increase of numbers and striving for patronage) have been too indifferent about qualifications and right preparation of young graduates to commence practice, and have graduated and turned loose upon communities incompetent men, some that never can practise with skill and credit to the profession and justice to the public. But such cases proportionately are small, unquestionably the exception, and not the rule, —a feature in all professions, and not more frequent or common with dentistry than medicine, law, and the sacred ministry; none exempt.

We must not be too exacting. Let us try to be charitable, generous, and hopeful, trusting strength may come of weakness, good of evil, and if we fail occasionally to realize the results desired and hoped for, we will be sustained through the satisfaction of knowing we erred on the side of charity and humanity, with good intentions for general good.

Examining boards seem to lose sight of the fact that when they reject and stamp an applicant for license to practise as incompetent and unworthy, it is a life-mark and stigma that time will not erase, and often with sensitive natures will be keenly felt through life.

It is possible that many young men of true worth may feel the sting of injustice so severely as to be deterred from making further effort, who, if favored and encouraged, as they should be, by older men in the profession, would in a few years become skilful operators, and truly merit confidence and praise as trustworthy practitioners.

In our zeal and ambition for a higher order of things (professional), we must not lose sight of the experiences of the past. Evolution is safer and grander for profitable and enduring results than revolution.

We, as a profession, are considerably presumptuous when we assume to contend for an advance and higher grade of attainments, skill, and practical excellence to begin with, than medicine, law, and the ministry. It is commendable to be ambitious, but it is wisest and best not to make haste to get ahead too fast.

Just think of the many men recorded, and some we have known and are now living, when commencing the practice of dentistry, medicine, law, and the ministry, what weakness, seemingly, and what little hope for them, and what a poor prospect for good to mankind through their labors; but time developed that there was will-power, manhood, and tact, backed by ambition and perseverance, which are moving factors in life-work that will accomplish much, and developments that will surprise and delight us.

Dental colleges should prepare students for graduation and to be able to begin practice. Time, application, and experience will develop and determine the possibility of attainments and manipulative ability.

We have no right to demand of colleges that every graduate just possessed of his diploma shall be a skilful operator or manipulator in any branch of the profession and well versed in the theory of dental science, and able to treat diseases and perform all operations within the range of his professional province as creditably and satisfactorily as after a five years' experience. Such a demand is unreasonable and unjust.

I will suggest to examining boards, and all who advocate their remaining longer than there is necessity for their existence, this fact, that many men now engaged in successful practice of dentistry and medicine, and are acknowledged to be first-class practitioners, are men whose advantages for preliminary education were extremely limited, and the advantages afforded them at dental and medical colleges very meagre compared to those of the present day.

But such men, often friendless and cramped by poverty, were richly endowed with will- and brain-power, and said silently to themselves in their struggle for advancement and usefulness, All I ask for and want is a fair chance and a fair race. It was granted, and should be to all such now, and victory and enviable reputation has been the reward.

Higher education is desirable and is needed on every line of life action, and it is coming quite as rapidly as will be for the good of a people. In educational matters, as in other affairs of life, slow progress, but steady and sure, is wisest and best. In climbing the ladder of life, professional or otherwise, one round at a time is safest and surest for reaching the top successfully.

We realize from observation a developed and unquestionable fact, that it is not always the case that the best educated men are the most progressive and useful and most successful, as a whole, in professions or any vocation, and are never the most inventive and faithful, persistent workers. Examining boards are clamoring for too much rapid advance at short notice, some of them forgetting their misfortune and needs in early life, and their very great disadvantages in the beginning of their early professional career.

Thorough professional knowledge and excellence in manipulative skill is not the work of a few months or years. Preparation to commence the effort for those higher and desired attainments is all that should be required or expected of young men who have just graduated and are ready to commence practice.

This great country of ours is a republic. We boast of it, and are proud of her institutions and the guaranteed equality of rights. If we are honest in our professions, we must sustain and perpetuate them in principle and purity, and must do nothing through false ideas and false ambition and pride to assist and elevate one class and lower and depress another.

The time has not yet arrived, but is far distant, I hope, when (if ever) in this blessed land none but the fortunate and specially favored need hope for and anticipate admittance to membership and honors in the dental profession. Should such a state of things ever occur, then there will be rapid deterioration, and dentistry as a profession will tend downward rather than upward and onward.

For progress, strength, and highest order of attainments all must feel and fully realize the equality of right and privilege to make effort and strive for the highest mark and prize attainable, and that the race is open to all, and there will be no prejudices of favoritism indulged, to the disfavor or favor of any. With such provisions of privilege, the stimulus will be great, and the competition and nobleness of struggle and effort put forth will be more exalted and meritorious and humanity better served; professional men will think better of themselves and will be better thought of. We must be just, humane, and honest, and lose sight of self as best we can when acting "in the interest of the public."

Education in our country is on the increase, and in every section provision is being made for higher grades for all classes, and good will come of it; but for best effect the process must be slow, the slower in reason the better, that all may enjoy equal chances for benefit and the whole be blessed thereby.

In place of examining boards I will suggest that each State create a board of censors, to whom shall be reported any and all cases of malpractice or improper professional conduct, and before whom the accused may and shall confront his accusers, and, if convicted, shall be suspended or barred the privilege to practise in the State in which the offence was committed; and the secretary of the board of censors shall be required to report proceedings and ruling, with seal of office, to every other board in the several States, who shall place on file and furnish copy to secretary of State dental association or society, to be read at ensuing meeting. By such a procedure the unworthy could be rightly dealt with and the public protected more securely and the profession sustained better than by the present provision and arrangement of examining boards.

B. F. ARRINGTON.

GOLDSBORO, N. C.

